# Artificial intelligence and labor demand: An empirical analysis of Chinese small and micro enterprises

**DOI:** 10.1016/j.heliyon.2024.e33893

**Published:** 2024-06-29

**Authors:** Gan Xu, Yue Qiu, Jingyu Qi

**Affiliations:** a School of Finance, Capital University of Economics and Business, Beijing, 100070, China; b School of Labor Economics, Capital University of Economics and Business, Beijing, 100070, China

**Keywords:** AI inputs, SMEs, Labor demand, Substitution effect, Creation effect

## Abstract

The widespread application of artificial intelligence (AI) technology has triggered a significant transformation in the economic structure and has brought profound changes to human society. As China promotes the digital transformation of industries, understanding how the investment in AI by small and micro enterprises (SMEs) affects labor demand, which is inextricably linked to “stable employment”, becomes an important question. This paper uses special data from 127 SMEs in 14 provinces from 2016 to 2020 and employs a two-way fixed effects model to study the impact of AI inputs on enterprises’ labor demand. The empirical results show that the impact of AI inputs on the labor demand of SMEs is not significant overall, but shows a significant negative effect in non-state-owned enterprises, private enterprises, and high-tech enterprises. There is a significant difference in the impact of AI inputs on the labor demand of different industries, with only the wholesale and retail industry demonstrating a significant positive impact. From the results of mechanism analysis, the substitution effect and creation effect of AI inputs on labor demand coexist, and in general, these two effects cancel each other out. However, the substitution effect dominates in some types of enterprises and industries. Finally, this paper discusses the government and enterprise coping strategies for the employment impact of AI applications based on empirical evidence and research results. This paper not only theoretically demonstrates that the impact of AI investment on firms' labor demand is uncertain, but also empirically demonstrates that Chinese firms' AI investment does not significantly affect firms' overall labor demand. This facilitates the government and enterprises to formulate strategies that can enhance the level of enterprise intelligence without impacting the labor market.

## 1 Introduction

In recent years, the deployment of AI technology within organizations has become increasingly prevalent. McKinsey & Company forecasted that by 2021, over half of global enterprises would have integrated at least one AI function, positively influencing their revenue and expenditure. Furthermore, it was projected that two-thirds of these enterprises would sustain their AI investment over the subsequent three years. The Shenzhen AI Industry Association, in its 2021 AI Development White Paper, highlighted that in 2020, the scale of China's core AI industry reached 325.1 billion yuan. The financing amount in the AI field was 89.62 billion yuan, and the number of AI-related enterprises totaled 6,425, with 59.1 % of these concentrated in the application layer.

The extensive application of AI technology is poised to instigate a significant shift in the economic structure, profoundly altering human production and lifestyle, modes of thinking, and effectuating a substantial leap in social productivity. Concurrently, the impact of AI applications on employment has garnered increasing attention from scholars. Frey and Osborne (2017) were the first to apply a probabilistic classification model to the O*NET^1^ database to estimate the likelihood of 702 occupations in the United States being supplanted by computers in the future [1]. Their findings suggested that 47 % of positions in the United States were at risk of automation. This study catalyzed global scholars to evaluate the rate of occupational substitution in their respective countries. Oschinski and Wyonch (2017) examined the Canadian labor market and found that the Canadian labor force in industries susceptible to automation constituted only 1.7 % of total employment [2]. They found no evidence to suggest that automation would lead to significant unemployment in the short term. David (2017) discovered that 55 % of occupations in Japan could be replaced by computers, with no significant disparity among workers of different genders [3]. Liu et al. (2022) determined that approximately 33 % of the labor force in China is in the high-risk category, with gender, age, education level, and wages all significantly correlated with the likelihood of replacement [4].

The digital transformation of enterprises will engender increased demand for AI, while simultaneously providing the fundamental conditions for AI application. With the ongoing innovation and development of various AI technology segments, which will bring about substantial changes in production and economic growth, enterprises will expand the scale of AI resource introduction, increase investment in independent research and development, integrate AI with their main business, and enhance their industrial status and core competitiveness (Liu et al., 2023) [5]. In this context, effectively coordinating the inherent conflict between intelligent manufacturing and labor employment has profound implications for both employment stability and digital transformation and upgrading in China in the new era.

The study shows that the impact of AI on the labor demand of enterprises mainly exists in the following effects: (1) substitution effect, i.e., the application of AI leads to a decline in employment; (2) creation effect, i.e., the application of AI creates new jobs and increases employment; and (3) combined effect, i.e., the employment creation effect of the application of AI offsets the substitution effect, and the overall impact is not significant. This paper applies the data of Chinese SMEs to test these effects separately.

This paper aims to examine the impact of AI inputs on the labor demand of Chinese SMEs and its mechanism of action. The study is based on research data from 127 SMEs across 14 provinces in China between 2016 and 2020. The results of the study show that: (1) From the perspective of total labor demand, the increase in AI inputs does not significantly affect the labor demand of SMEs. Although the benchmark regression results indicate that AI inputs do not have a significant impact on the total labor demand in enterprises, the regression results suggest a potential substitution effect of AI inputs on labor demand. Specifically, every 10 % increase in AI inputs results in approximately a 0.2 % decrease in labor demand. Concurrently, the scale of the enterprise's output has a positive impact, aligning with the conclusions of the theoretical model analysis, albeit the coefficients are not significant. The average wage level of enterprise employees has a significant negative impact, which is consistent with the findings of Li et al. (2021) [6]. (2) Further analysis reveals the existence of both substitution and creation effects of AI inputs on enterprise labor demand. The impact of AI inputs on the total labor demand in enterprises stems from the substitution effect on low-skilled labor and the creation effect of AI-related labor offsetting each other. This results in the insignificant impact of AI inputs on the total labor demand in enterprises. (3) The impact of AI inputs on the labor demand of Chinese SMEs exhibits heterogeneity among industries or types of enterprises. In the wholesale and retail industry, AI inputs have a significant creation effect on the total labor demand of enterprises. However, in the manufacturing industry and other service industries, the impact of AI inputs on the total labor demand of enterprises is not significant.

The marginal contributions of this paper, in comparison to existing studies, are as follows: Firstly, while most existing research on AI and firms' labor demand relies on data such as the installed base of industrial robots (Graetz and Michaels 2015; Südekum et al., 2017; Acemoglu and Restrepo 2018) [[7], [8], [9]], the number of imported industrial robots (Li et al., 2021) [10], and the number of AI patents (Hoedemakers, 2017) [11], this paper takes a different approach. Recognizing that AI technology applications in non-manufacturing firms may no longer be in the form of industrial robots (e.g., fingerprint identification, smart retailing, etc.), this paper measures firms' AI investment in terms of the amount of AI investment. This approach encompasses a wider range of AI-applying firms and avoids omitting a significant number of firms with non-robotics applications. Secondly, the existing literature primarily utilizes data from listed or state-owned enterprises for analysis. This approach can obscure the impact of AI applications on all market participants, especially given that the vast majority of China's market players are small and micro enterprises (accounting for 90 % of the total). These enterprises are characterized by significant financing constraints and difficulties in technological innovation compared to large enterprises. Therefore, the impact of AI applications on the labor demand of SMEs may yield different results. This paper shows that the impact of AI applications on the total labor demand of SMEs is not significant, which contrasts with the negative impact found by Wang and Dong (2020) [12] and the positive impact found by Li et al. (2021) [10]. It also differs from Südekum et al. (2017) [8], which found that AI reduces employment in the manufacturing industry while increasing employment in the service industry, with the impacts of both effects offsetting each other, resulting in a non-significant overall impact. Thirdly, this paper tests multiple impact channels. It examines a variety of possible channels through which AI inputs affect the labor demand of SMEs, including the substitution effect, the complementary effect, and the creation effect caused by AI applications. Fourthly, to ensure the accuracy of identification, this paper adopts multiple causal identification strategies. The 2SLS + IV method, SYS-GMM, and lag term regression are used to control the endogeneity problem of AI application, ensuring the robustness of the analytical conclusions.

The structure of this paper is organized as follows: the second part is the literature review; the third part is the theoretical model analysis; the fourth part is the model setting and data description; the fifth part is the empirical analysis, including the benchmark regression results, the endogeneity test, and the robustness test; the sixth part is the further analysis, including the analysis of heterogeneity and the analysis of the mechanism, which explores the role of the pathway of AI inputs in affecting the demand for labor; and the seventh part is the conclusion and recommendations.

## 2 Literature review

The advent of AI has sparked discussions on the impact of this new technology on enterprise labor demand. Scholars worldwide have presented diverse results through theoretical and empirical research. The influence of AI on enterprise labor demand primarily manifests as substitution effects, creation effects, and comprehensive effects. This paper will elaborate on the impact of AI on enterprise labor demand from these three perspectives.

### 2.1 Substitution effect of AI on enterprise labor demand

Acemoglu and Restrepo (2018) analyzed the impact of the increased use of robots on the local labor market in the U.S. over the period 1990–2007 [7]. Utilizing the IFR and EU KLEMS (EU Capital, Labor, Energy, Materials, and Services) datasets, their results indicated that the use of robots indeed reduces employment. For every 1000 people added to the population, the proportion of the employed population decreases by approximately 0.18 %–0.34 %. AI applications reduce firms' demand for low-skilled labor, leading to a decrease in the total demand for labor by firms (Brynjolfsson and Mitchell, 2017) [13]. Weiss et al. (2022) experimentally investigated how applicants' use of AI affects their chances of getting a job. The results of the different experiments showed that the use of AI technology can be perceived negatively [14]. Khaliq et al. (2022), by studying 330 employees (managers, supervisors, front desk and room attendants) working in three and five-star hotels in Lahore, Pakistan, showed that AI and Robot Awareness had a significant positive relationship with employees’ tendency to leave their jobs [15]. Therefore, this paper proposes:

Hypothesis 1 AI inputs have a negative effect in specific enterprises.

### 2.2 Creation effect of AI on enterprises’ labor demand

Technological change increases the number of non-conventional occupations in developed and emerging countries (Reijnders and Vries, 2018) [16]. Among other things, disembodied technological change has a positive impact on employment dynamics in “upstream” industries, as well as expansionary investments in “downstream” industries (Dosi et al., 2021) [17]. Damioli et al. (2023) conducted an empirical analysis based on a global longitudinal sample of more than 3500 leading firms that filed patents for AI-related inventions in the period 2000–2016 (obtained by merging the EPO, PATSTAT and BvD-ORBIS databases) [18]. The results show that AI product innovations increase employment opportunities. Therefore, this paper proposes:

Hypothesis 2 AI inputs have a positive effect in specific enterprises.

### 2.3 Combined effect of AI on enterprises’ labor demand

AI exerts a comprehensive effect on firms' labor demand, encompassing both substitution and creation effects. The substitution effect reduces firms’ labor demand, but the application of AI increases the productivity of automated tasks while also increasing the demand for labor for non-automated tasks (Acemoglu and Restrepo, 2018) [19]. Graetz and Michaels (2015) utilized IFR panel data for 17 countries over the period 1993–2007 and found no significant impact of industrial robots on total employment [9]. Südekum et al. (2017), using IFR data for Germany over the period 1994–2014, found that robot use did not result in an overall loss of employment (This accounts for almost 23 % of the overall decline of manufacturing employment in Germany over the period 1994–2014, roughly 275,000 jobs. But this loss was fully offset by additional jobs in the service sector.), but merely altered the composition of employment in Germany. Specifically, while robot use reduced employment in manufacturing, it increased employment in the service sector [8]. Hunt et al. (2022) proposed a new methodology based on a customized employer survey, which showed that organizations that introduced AI had higher rates of both job creation and job destruction compared to organizations that introduced non-AI technologies [20]. Their findings suggest that job creation is as likely as job destruction, which may alleviate concerns about AI in the workplace. Fossen and Sorgner (2022) examined the heterogeneous impact of new digital technologies on individual-level wage and employment dynamics in the United States over the period 2011–2018, based on panel data from the CPS and ASEC [21]. Their results show that digital technologies that displace the labor force are associated with slower wage growth and higher probabilities of switching occupations and non-employment. In contrast, digital technologies that restore the labor force improve individual labor market outcomes. Workers with high levels of formal education are most affected by the new generation of digital technologies. Therefore, this paper proposes:

Hypothesis 3 In whole, AI inputs have an insignificant effect.

### 2.4 Impact of AI application on labor demand of Chinese companies

Firstly, the impact of AI applications on employment is not significant. Currently, China's AI is in the weak AI development stage and technology growth period (see Table 1). The destructive effect of AI applications on employment is limited, but the long-term employment effect is not optimistic (Wang et al., 2017; Zhang et al., 2021) [12,22]. The change in employment numbers due to AI depends on the relative size of the creation effect and the destruction effect (Tang and Zhang, 2020) [23]. The development of AI and the improvement of technology level will increase the relative supply of skilled and unskilled labor, contributing to the improvement of the overall quality of the workforce and the optimization of the workforce structure (Zhu and Li, 2018) [24]. In the advancement of AI and automation, the total amount of employment will remain basically stable under the substitution effect and inhibition effect, but structural shocks are inevitable. Middle-level positions are easily replaced, and the employment structure will show a polarization trend (Wang et al., 2020, Cai and Chen, 2019; Hui and Jiang, 2020) [12,25,26]. Secondly, AI applications have a substitution effect on employment. A large number of production personnel in the primary and secondary industries and a large number of employed people in the wholesale and retail trade, finance, transportation, storage and postal services in the tertiary industry, as well as accounting, statistics, auditing, and administrative and logistical personnel in enterprises, institutions, and agencies, will be replaced by AI in a large number of cases (Chen, 2019) [27].

**Table 1:** Metrics for various drivers in China's AI development.

Main Driver in AI	Proxy Measure(s)	China	USA
Hardware	Int'l market share of semiconductor prod. (2015)	4 % of the world	50 % of the world
	Financing for FPGA chip makers (2017)	USD 34.4 million (7.6 % of world)	USD 192.5 million (42.4 % of world)
Research and Algorithms	Number of AI experts	39, 200 (13.1 % of world)	78,700 (26.2 % of world)
	Percentage of AAAI Conference Presentations (2015)	20.5 % of world	48.4 % of world
Commercial AI Sector	Proportion of world's AI companies (2017)	23 %	42 %
	Total investments in AI companies (2012–2016)	USD 2.6 billion (6.6 % of world)	USD 17.2 billion (43.4 %)

The potential risk of replacement varies across occupations, with research, socializing, and management occupations having a lower potential risk of replacement. Occupations with more procedural cognitive activities, such as clerical and reviewing occupations, have a higher potential risk of replacement. It is estimated that about 59.5 % of Chinese jobs will be impacted by AI in the next 20 years (Gong and Peng, 2020) [28]. In the short term, complex, general-purpose, non-repetitive labor that requires full civil responsibility is difficult to replace. In the long term, labor that requires causal reasoning is difficult to replace. AI can never replace inventive, emotional, artistic, and other aspects of labor, which constitute the limit of labor replacement by AI (Cheng, 2020) [29]. The short-term impact of technological advances in AI on labor employment fluctuates greatly, and in the long term, it will lead to a reduction in employment (Han and Han, 2020) [30]. The integration of AI in manufacturing enterprises significantly reduces the share of low-skilled employment (Xie et al., 2020; Yan et al., 2020) [31,32], and at the same time increases the share of employment in the service industry, especially the knowledge- and technology-intensive modern service industry. This promotes the seniorization of the employment structure of the industry and helps to achieve high-quality employment (Wang, 2020) [33]. The expansion of the scale of robot application will significantly reduce the employment level of the local labor force in the coming year, especially the employment level of industries that are easily replaced by machines (Kong et al., 2020) [34]. Robot application has a certain substitution effect on the labor demand of enterprises (Wang and Dong, 2020) [12]. Intelligence has a significant substitution effect on labor employment in China, reducing the growth of employment on the one hand, but increasing the working hours of the active labor force on the other (Zhou et al., 2021) [35]. In addition, the impact of AI on employment has an antipolar phenomenon, that is, AI technology is able to replace complex labor, and the impact on employment is manifested in a significant increase in the proportion of medium-skilled workers in employment, and a decrease in the proportion of high-skilled and low-skilled workers in employment. Third, AI applications have a creation effect on employment. The investment in AI technology research and development leads to the expansion of the number of jobs, i.e., the size of employees (He and Qiu, 2020) [36]. The productivity released by robots can create more jobs to absorb the influx of immigrants (Wei et al., 2020) [37]. AI substitution promotes the outflow of human capital from the manufacturing industry and the inflow of human capital from the service industry, and promotes the enhancement of human capital in terms of education and comprehensive power, and the enhancement of jobs and wage incomes (Tan and Zhang, 2021) [38]. With the increase in the use of industrial robots, the employment scale of high-skilled labor rises, resulting in the “compensation effect”, and the employment scale of low-skilled labor falls, resulting in the “substitution effect”, while the impact on middle-skilled labor is not significant. Therefore, this paper proposes:

Hypothesis 4 The impact of AI inputs on the labor demand of Chinese SMEs is not significant, but there are industry and firm heterogeneities.

## 3 Theoretical model analysis

This paper utilizes the task-based theoretical framework of Acemoglu and Restrepo (2016, 2018, 2020) to analyze the impact of AI inputs on enterprises’ labor demand [19,39,40].

Assuming that enterprises’ production function takes the form of a Cobb-Douglas production function, each enterprise produces output by combining capital with successive tasks with (*s* ∈ [0,1]), each of which can be produced using either AI technology and equipment or human labor. We denote by y_it_ (s) the number of tasks (s) used in the production of (Y_it_). These tasks are combined in a fixed ratio, i.e.,

(1) $$\begin{array}{c} Y_{\text{it}}=A_{\text{it}}{\left[\min_{s\in \left[0,1\right]}\left\{y_{\text{it}}\left(s\right)\right\}\right]}^{\alpha }K_{\text{it}}^{1-\alpha },\alpha \in \left(0,1\right) \end{array}$$

where Y_it_ is the total output of enterprise (i) in year (t), y_it_(s) is the output of task (s), A_it_ is the average technology level, K_it_ includes traditional physical capital as well as AI inputs, and (1-α) is the output share of capital.

The AI technology replaces workers in some of the previous tasks. Specifically, in enterprise (i), tasks ([0, θi]) are technologically automated and can be performed by AI. Assuming that all enterprises have access to the same technology, the output of task (s) in enterprise (i) is:

(2) $$\begin{array}{c} y_{\text{it}}\left(s\right)=\left\{ \begin{array}{c} \sigma_{L}l_{\text{it}}\left(s\right)+\sigma_{a}m_{\text{it}}\left(s\right),s\in \left[0,\theta i\right]\\ \sigma_{L}l_{\text{it}}\left(s\right),s\in \left[\theta_{i},1\right] \end{array}\right. \end{array}$$

where α_L_ is the productivity of labor in task (s), α_a_ is the productivity of AI technology, the labor input of task (s) is l_it_(s), the AI input is m_it_ (s).

Based on the principle of enterprise cost minimization, this paper obtains the labor demand for task (s) in year (t) of enterprise (i), i.e.,

(3) $$\begin{array}{c} l_{\text{it}}\left(s\right)=\left\{ \begin{array}{c} \;0,s\in \left[0,\theta i\right]\\ {\left(\frac{Y_{\text{it}}}{A_{\text{it}}K_{\text{it}}^{1-\alpha }\sigma_{L}^{\alpha }}\right)}^{\frac{1}{\alpha }},s\in \left[\theta i,1\right] \end{array}\right. \end{array}$$

Since task (s) is a continuous task, the total labor demand of enterprise (i) in year (t) is:

(4) $$\begin{array}{c} L_{\text{it}}=\int_{0}^{\theta_{i}}l_{\text{it}}\left(s\right)d_{s}+\int_{\theta_{i}}^{1}l_{\text{it}}\left(s\right)d_{s} \end{array}$$

Substituting equation (3) into equation (4) yields that：

(5) $$\begin{array}{c} L_{\text{it}}=\left(1-\theta_{i}\right){\left(\frac{Y_{\text{it}}}{A_{\text{it}}K_{\text{it}}^{1-\alpha }\sigma_{L}^{\alpha }}\right)}^{\frac{1}{\alpha }} \end{array}$$

A log-linearized transformation of equation (5) yields:

(6) $$\begin{array}{c} \ln\;L_{\text{it}}=\ln\left(1-\theta_{i}\right)+\frac{1}{\alpha }\ln\;Y_{\text{it}}-\frac{1}{\alpha }\ln\;A_{\text{it}}-\frac{1-\alpha }{\alpha }\ln\;K_{\text{it}}-\ln\;\sigma_{L} \end{array}$$

**Fig. 1:**
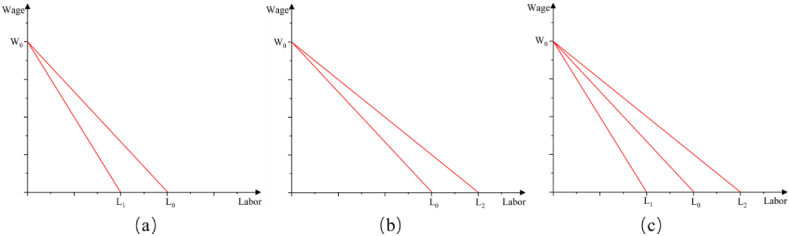
The impact of AI on labor demand, (a) substitution effect, as the increase of (θi, K_it_, A_it_) will lead to the tasks originally done by labor to be replaced by AI, labor demand will decline; (b) creation effect, as the increase of Y_it_, AI improves enterprises' productivity, labor demand will rise; (c) combined effect, AI adoption causes substitution effect in some industries, and creation effect in other industries, that is combined effect of these two forces depends on which one dominates. In Fig. 1, L_0_ indicates labor demand before AI adoption, L_1_ shows labor demand resulted in substitution effect, L_2_ means labor demand creation effect by AI; W_0_ indicates average wage, Due to the existence of wage stickiness, this paper assumes that average wages remain constant in the short run.

According to the results of equation (5), it can be found that the impact of enterprise application of AI technology on labor demand is mainly reflected in two aspects: first, the substitution effect. Because the increase of (θi) will lead to the tasks originally done by labor to be replaced by AI, resulting in the reduction of enterprise labor demand (see Fig. 1 (a), labor demand decline from L_0_ to L_1_), this reduction may be the share of labor being intelligent, or it may be the reduction of the total demand for enterprise labor. However, State-owned enterprises are the “ballast” of social stability, assuming a large number of social functions and responsibilities, providing a large number of jobs, and maintaining the political and social stability of the country. Therefore, state-owned enterprises labor demand is not significantly affected by AI; Second, the creation effect (or productivity effect). The application of AI technology improves enterprise's productivity, resulting in an increase in the size of enterprises' output (Y_it_), which increases enterprises' demand for labor in non-intelligent jobs (Acemoglu and Restrepo, 2018) [7] (see Fig. 1 (b), labor demand increase from L_0_ to L_2_). In fact, data from the National Bureau of Statistics (NBS) shows the year-end workforce in the wholesale and retail industry growing from 11.936 million in 2016 to 12.35 million in 2020. In addition, an increase in AI inputs (K_it_) can also directly reduce enterprises' demand for labor, so there is uncertainty about the impact of AI inputs on enterprises' demand for labor (see Fig. 1 (c), if (L_0_-L_1_) > (L_2_-L_0_), means combined effect is substitution effect; if (L_0_-L_1_) < (L_2_-L_0_), means combined effect is creation effect; if (L_0_-L_1_) = (L_2_-L_0_), means combined effect is substitution effect).

## 4 Data description and model setup

### 4.1 Data description

This paper utilizes the random sampling method to select 127 enterprises nationwide for a questionnaire survey, covering 14 provinces, and obtains a total of 635 observations. Among them, the registered capital data are obtained from the website of the National Enterprise Credit Information Publicity System, and the accuracy of the basic information of the enterprises is verified at the same time of obtaining the registered capital data. It is found that all the basic information of the enterprises in all the questionnaires is correct after comparison, which indirectly ensures the reliability of the data in the questionnaires. In addition, the definition of SMEs in this paper comes from the National Individual Private Economic Development Service Network (micro and small enterprise directory) as well as from the website of TIANYANCHA.^2^ As the whole survey process has been strongly supported by relevant organizations, the interviewed enterprises can better cooperate with the survey, reduce the refusal rate, and the quality of the questionnaire is also guaranteed. In the process of random sampling, the enterprises were firstly selected from the sampling frame, and then one enterprise manager and/or 1–2 financial managers were selected from the selected enterprises. The sample enterprises were distributed across 16 broad industry categories, including information transmission, software and information technology services, manufacturing, wholesale and retail trade, transportation, warehousing and postal service, accommodation and catering, and education, and covered five types of enterprise ownership (state-owned enterprises, limited liability companies, foreign-invested enterprises, wholly owned individual enterprises, and private enterprises).

### 4.2 Model setting

In this paper, to examine the impact of AI inputs on the labor demand of SMEs, the benchmark regression model of this paper is obtained according to equation (6) in the theoretical model analysis, that is two-way fixed effects model (see equation (7)). Two-way fixed effects introduce both individual and time-specific dummy variables to control for characteristics or influences that are fixed across individuals and time. Eliminating individual and time fixed effects allows for more accurate estimation of the effects of other explanatory variables on the explanatory variables and avoids the perturbation of the estimation results by individual and time heterogeneity; controlling for individual and time fixed effects focuses more on the causal relationship between explanatory variables rather than on individual or time-specific effects. Overall, the individual and time two-way fixed effects model helps to reduce bias and improve estimation accuracy in panel data analysis, allowing researchers to explore the relationships between variables in economics in greater depth.

(7) $$\begin{array}{c} \ln\;L_{\text{it}}=\beta_{0}+\beta_{1}{{\text{lnAI}}_{\text{inv}}}_{\text{it}}+\beta C_{\text{it}}+\mu_{i}+\delta_{t}+\lambda_{\text{it}} \end{array}$$

where L_it_ denotes the total labor demand of enterprise (i) in year (t), measured by the natural logarithm of the total number of employees of the enterprise; ln AIinv_it_ denotes the AI input of enterprise (i) in year (t), measured by the natural logarithm of the investment in the enterprise's AI technology, which is the core explanatory variable in this paper, and (β_1_ < 0) from the analysis of the theoretical model; C_it_ denotes the control variables of the benchmark model, with reference to Mao and Xu (2016), Li et al. (2021), and Wang et al. (2021), the control variables include [[41], [42], [43]]:

1. The age of the enterprise (age) and its square term (age^2^);
2. The performance of the enterprise (or the size of the output that (ln income)), the performance of enterprise (i) in year (t) is the natural logarithm of the enterprise's main business revenue;
3. R&D investment (ln rd), the R&D investment of enterprise (i) in year (t) is the natural logarithm of the enterprise's R&D expenses;
4. Average wage level of the enterprise (ln pwage), the average wage level of enterprise (i) in year (t) is the natural logarithm of the enterprise's total wage bill divided by the total number of employees.

The details are shown in Table 2. In addition, this paper also adds dummy variables for enterprise type (state-owned enterprises, private enterprises, and high-tech enterprises) to control for the baseline model, respectively, and controls for the fixed effects of individual enterprise (μ_i_), year (δ_t_), industry, city, and industry*year; and (λ_it_) is a random disturbance term.

**Table 2:** Descriptive statistics.

Symbol	Observations	Mean	Standard Deviation	Minimum	Maximum
ln staff	635	4.555	1.549	0.693	9.680
ln low	635	3.377	1.967	0	9.306
ln ug	635	3.740	1.665	0	9.393
ln AIinv	635	4.278	2.629	0	15.607
age	635	10.457	7.728	0	56
ln income	635	7.885	2.413	0.140	18.198
ln rd	635	4.563	2.587	0	16.811
ln pwage	635	1.914	1.395	0.041	12.388

## 5 Empirical analysis

### 5.1 Benchmark regression results

Table 3 reports the benchmark regression results. Columns (1)–(5) show the regression results of the two-way fixed effects model with control variables added sequentially, and column (6) shows the regression results of the random effects model. The Hausman test of the regression results in columns (5) and (6) shows that the fixed effects model is more reasonable, so the following models in this paper are built on the basis of the fixed effects model. The results in columns (1)–(5) of Table 3 show that there is a negative correlation between AI inputs and total demand for labor in enterprises, but the coefficient of AI inputs is not significant, and the significance of the coefficients of AI inputs and the robust standard error do not change significantly when different control variables are added, which suggests that the baseline model has better robustness.

**Table 3:** Impact of AI inputs on aggregate labor demand.

Variables	(1)	(2)	(3)	(4)	(5)	(6)
ln AIinv	−0.009 (0.016)	−0.009 (0.015)	−0.017 (0.015)	−0.023 (0.016)	−0.020 (0.015)	−0.012 (0.015)
age		0.103*** (0.027)	0.081** (0.031)	0.079** (0.030)	0.091*** (0.030)	0.110*** (0.026)
age2		−0.002* (0.001)	−0.002** (0.001)	−0.002** (0.001)	−0.002** (0.001)	−0.001 (0.001)
ln income			0.144 (0.103)	0.140 (0.101)	0.158 (0.109)	0.193* (0.107)
ln rd				0.025 (0.032)	0.033 (0.030)	0.041 (0.029)
ln pwage					−0.225* (0.125)	−0.250*** (0.091)
cons_	4.436*** (0.016)	3.840*** (0.153)	2.950*** (0.642)	2.928*** (0.629)	3.066*** (0.684)	2.511*** (0.710)
Hausman -P					0.000	
Enterprise fixed	YES	YES	YES	YES	YES	YES
Year fixed	YES	YES	YES	YES	YES	YES
Observations	635	635	635	635	635	635
F value	5.400***	4.960***	5.250***	4.830***	7.250***	115.680***
R^2^	0.116	0.150	0.225	0.229	0.258	0.247

Note: Robust standard errors in parentheses, standard errors clustered at the enterprise level, *, **, and *** indicate significance at the 10 %, 5 %, and 1 % levels, respectively. Same below.

The benchmark regression results presented in Table 3 suggest that investment in AI does not significantly influence the overall employment demand within enterprises. This observation, to some extent, resonates with the findings of existing research. Numerous studies have demonstrated that under the influence of AI, the substitution and creation effects of employment interact (Wang et al., 2017; Zhang et al., 2021) [12,22]. At the enterprise level, the demand for and application of AI varies across different positions. Consequently, an increase in AI investment may simultaneously replace certain roles within the enterprise, such as physical labor (Brynjolfsson and Mitchell, 2017) [13], while creating demand for new positions, such as social work (Dosi et al., 2021, Damioli et al., 2023) [17,18]. Given that the demand for AI across different types of labor depends on the relative impact of these two effects (Acemoglu & Restrepo, 2019a), the overall impact of AI input on labor demand remains uncertain (Dauth et al., 2017) [8].

This paper suggests that the reason for the non-significant impact of AI inputs on the aggregate demand for labor in SMEs may be that the use of AI technology substitutes for low-skilled labor while at the same time increasing the demand for AI-related labor. This hypothesis will be argued in detail in the further analysis section. Additionally, we examine labor demand across various job types to ascertain the impact of AI investment on the total labor demand of enterprises. Survey results indicate that the increase in technical or R&D positions and sales positions significantly outweighs the decrease. Conversely, the decrease in human or administrative management positions, product production or service provision positions, and customer service positions is greater than the increase. These findings suggest that increased AI investment by small and micro enterprises will have a differentiated impact on labor demand across different positions, thereby resulting in an insignificant impact on the total employment demand of the enterprise. Concurrently, the impact of other control variables on the total employment demand of the enterprise aligns with existing research, such as the influence of enterprise age and its square term on the total employment demand (Li Lei et al., 2021) [6].

### 5.2 Endogeneity test

To address the estimation bias caused by the endogeneity problem, this paper combines the main reasons that cause the endogeneity problem with the data characteristics, and focuses on the endogeneity problem from two main aspects, i.e., omitted variables and mutual causation.

To mitigate the impact of omitted variables, such as capital intensity (Wang et al., 2021) [43], on the benchmark regression results, this paper adopts the instrumental variable method to estimate the benchmark regression model with 2SLS and SYS-GMM, respectively, and explores the endogeneity problem caused by omitted variables. Drawing on Liu et al. (2022) [4], the lagged period of AI inputs is used as an instrumental variable for the 2SLS and SYS-GMM models, and the regression results are shown in Table 4. In Table 4, column (1) shows the regression results of the first stage of 2SLS and column (2) shows the regression results of the second stage of 2SLS. Among them, the first-stage results show that AI input lagged one period is significantly positively correlated with AI input and there is no weak identification problem (Cragg-Donald Wald F-statistical value is greater than 10 % maximum critical value), indicating that the instrumental variables satisfy the correlation as well as exogenous; the second-stage results show that the negative effect of AI inputs on the total demand for labor in the enterprise is insignificant, and that the coefficients of AI inputs’ coefficients increase by about a factor of one over the benchmark regression results, indicating that the measurement error of AI inputs is not significant (Zhang et al., 2021), i.e., the benchmark regression results are robust [44].

**Table 4:** Results of endogeneity test.

Variables	2SLS + IV		SYS-GMM	Lagged	
	(1)	(2)	(3)	(4)	(5)
	lnAI_inv	lnstaff	lnstaff	lnstaff	lnAI_inv
lnAI_inv		−0.042 (0.035)	−0.010 (0.014)		
L.lnAI_inv	0.247*** (0.091)		−0.004 (0.008)	−0.010 (0.009)	
lnstaff					−0.299 (0.219)
L.lnstaff			0.487*** (0.060)		
Controls	YES	YES	YES	YES	YES
Cragg-Donald Wald F Values	34.179				
10 % maximum critical value	16.380				
AR（1）-P value			0.000		
AR（2）-P value			0.944		
Sargan -P value			0.270		
Observations	508	508	381	508	635
F value		8.090***	27.680***	4.350***	21.830***
R^2^		0.139	0.229	0.150	0.398

To avoid possible endogeneity problems caused by reverse causality, this paper uses the first-order lagged term of AI inputs for regression analysis (Zhang et al., 2021) [22]. The results in column (4) of Table 4 show that the negative effect of the first-order lagged term of AI inputs on the aggregate demand for labor in enterprises remains insignificant, indicating that the benchmark regression results are robust. In addition, this paper conducts a double fixed effects model regression with AI inputs as the explanatory variables and aggregate enterprise labor demand as the explanatory variables, and the results are shown in column (5) in Table 4, which indicate that the effect of aggregate enterprise labor demand on AI inputs is insignificant, i.e., there is no endogeneity problem of mutual causality between AI inputs and aggregate enterprise labor demand.

The results of the SYS-GMM model in column (3) of Table 4 show that the first-order lag term of the total demand for labor is significantly positively correlated with the total demand for labor, and neither the AI inputs nor its first-order lag term has a significant negative effect on the total demand for labor, indicating that the benchmark regression results are robust. The results of the 2SLS model as well as the systematic GMM model show that the regression results of the instrumental variables approach are consistent with the benchmark regression results, i.e., the AI inputs of SMEs are not significantly affected by the measurement error (Zhang et al., 2021), i.e., the benchmark regression results are robust [44].

### 5.3 Robustness tests

#### 5.3.1 Replacement variables

The R&D investment of AI-listed companies and the intensity of R&D investment are proxy variables for measuring AI investment (Huang et al., 2021) [45]. This paper regresses R&D investment as a proxy variable for AI investment on the total demand for labor in enterprises. The results are shown in Table 5. The results in column (1) of Table 5 show that there is a negative correlation between AI inputs and the total demand for labor in enterprises, but the coefficient of AI inputs is not significant. This is consistent with the results of the benchmark regression, indicating that the results of the benchmark regression are robust.

**Table 5:** Substituting variables and reducing sample size.

Variables	(1)	(2)	(3)	(4)	(5)
lnAI_inv	−0.018 (0.014)	−0.011 (0.013)	−0.013 (0.013)	−0.031 (0.023)	−0.011 (0.013)
Controls	YES	YES	YES	YES	YES
Enterprise fixed	YES	YES	YES	YES	YES
Year fixed	YES	YES	YES	YES	YES
Observations	635	635	596	254	381
F value	114.280***	133.590***	157.250***	5.930***	11.400***
R^2^	0.247	0.309	0.396	0.406	0.253

#### 5.3.2 Shrinking the sample size - excluding outliers

To prevent the influence of outliers, this paper shrinks the continuous variables at the upper and lower 1 % quantile (Cai et al., 2021) [46] as well as truncates them (Li et al., 2021) [41], and tests the robustness of the baseline regression through a two-way fixed effects model regression (as shown in Table 5). In particular, column (2) in Table 5 presents the regression results after truncating the sample (all values greater than the 99th percentile value are equal to the 99th percentile value, and all values less than the 1st percentile value are equal to the 1st percentile value), and the results show that the coefficients of the AI inputs are still insignificant and the coefficient sizes have not changed significantly. Column (3) in Table 5 shows the regression results after truncating the sample (all values greater than the 99 % quantile value are excluded and all values less than the 1 % quantile value are excluded) and the results show that the AI input coefficients are not significant. It shows that under the premise of considering the effect of outliers, the negative impact of AI input on the total demand for labor in enterprises is consistent with the results of the benchmark regression, indicating that the results of the benchmark regression are robust.

In 2017, the State Council issued the New Generation AI Development Plan, which points out that it accelerates the intelligent upgrading of industries. Therefore, this paper takes 2017 as the cut-off point and divides the entire sample into the 2016–2017 sub-sample and the 2018–2020 sub-sample for group regression to examine the robustness of the benchmark model, and columns (4) and (5) of Table 5 show the regression results of the two sub-samples respectively, which show that the negative impact of AI inputs on the total demand for labor in enterprises is not significant, indicating that the benchmark model regression results are robust.

#### 5.3.3 Controlling for industry, city, and other variables

In this paper, fixed effects for variables such as industry and city as well as industry × year fixed effects are added to the baseline regression model respectively to examine the robustness of the baseline regression results. Table 6 reports the test results controlling for variables such as industry and city. The regression results from Column (1) to Column (5) show that the coefficients of AI inputs remain insignificant after controlling for different dummy variables (individual, year, industry, city, and industry × year), indicating that the negative impact of AI inputs on the total demand for labor in enterprises is insignificant, i.e., the benchmark regression results are robust.

**Table 6:** Controlling for industry, city and other variables.

Variables	(1)	(2)	(3)	(4)	(5)
lnAI_inv	−0.019 (0.014)	−0.020 (0.015)	−0.020 (0.016)	−0.014 (0.015)	−0.011 (0.017)
Controls	YES	YES	YES	YES	YES
Enterprise fixed	YES	YES	YES		
Year fixed		YES	YES	YES	YES
Industry fixed			YES	YES	YES
City fixed				YES	YES
Industry × Year					YES
Observations	635	635	635	635	635
F value	7.760***	7.250***			
R^2^	0.248	0.258	0.258	0.251	0.382

## 6 Further analysis

### 6.1 Heterogeneity analysis

#### 6.1.1 Different enterprise ownership

The sample observation values reflected in the different nature of the enterprise may lead to biased conclusions. According to the nature of the enterprise, the sample enterprises are divided into non-state-owned enterprises and state-owned enterprises. The impact of AI inputs of SMEs on the total demand for labor in enterprises is analyzed through the two-way fixed effects model. The results are shown in Table 7. From the regression results in columns (1) and (2) of Tables 7 and it can be found that the coefficients of AI inputs of SMEs are significantly different between non-state enterprises and state-owned enterprises. In non-state enterprises, the coefficients of AI inputs are significantly negative at the 10 % level, while in state-owned enterprises, the coefficients of AI inputs are not significant. This shows that the impact of AI investment on enterprise labor demand is significantly different among enterprises of different natures, and the negative impact of artificial intelligence investment on enterprise labor demand is mainly concentrated in non-state-owned enterprises. The reason is that, on the one hand, compared with state-owned enterprises, non-state-owned enterprises are relatively weak in executing government policies and are slower in guiding government policies; on the other hand, the research and development of artificial intelligence technology by corporate technology R&D departments not only requires The investment in hardware requires high-end talents who master artificial intelligence technology, which increases the financial pressure and talent demand of enterprises (Rui Feng et al., 2024) [53]. Therefore, non-state-owned enterprises face many challenges in the process of increasing investment in artificial intelligence, and their ability to absorb high-skilled talents is weak, thus mainly showing a substitution effect on labor demand.

**Table 7:** Different enterprises’ type.

Variables	(1)	(2)	(3)	(4)	(5)	(6)
lnAI_inv	−0.027* (0.015)	0.003 (0.027)	0.040 (0.045)	−0.033** (0.014)	0.002 (0.017)	−0.058* (0.031)
Controls	YES	YES	YES	YES	YES	YES
Enterprise fixed	YES	YES	YES	YES	YES	YES
Year fixed	YES	YES	YES	YES	YES	YES
Observations	550	85	125	510	450	185
F value	8.050***	9.190***	2.950**	7.550***	5.550***	12.110***
R^2^	0.292	0.376	0.335	0.299	0.183	0.569

Since private enterprises are relatively more flexible and have more autonomy when choosing artificial intelligence technology, this study further explores the differential impact of artificial intelligence investment between private enterprises and non-private enterprises. Columns (3) and (4) in Table 7 show the regression results of the sample of non-private enterprises and the sample of private enterprises respectively. In the sample of non-private enterprises, the coefficient of AI input is positive but insignificant. In contrast, in the sample of private enterprises, the coefficient of AI input is significantly negative at the 5 % level, indicating that AI inputs have an inhibitory effect on the aggregate demand for labor in private enterprises. In addition, columns (5) and (6) in Table 7 conducted regression analyses for the non-high-tech enterprise sample and the high-tech enterprise sample, respectively. The results show that AI inputs have a significant inhibitory effect on the total demand for labor in high-tech enterprises, and the effect is not significant in the sample of non-high-tech enterprises. This indicates that the substitution effect of AI inputs is significant on the total demand for labor in technology-intensive enterprises.

#### 6.1.2 Different regions

Since there are certain differences in the economic development level, industrial structure and production technology level of various regions in China, the development trends between different regions are also obviously different (Hui and Jiang, 2020) [26]. To this end, we examined the regional heterogeneity of the impact of increased investment in artificial intelligence by small and micro enterprises on corporate labor demand. We draw on the classification method of Kong et al. (2020) [34], divides enterprises into enterprises in the eastern region and central and western regions, and performs group regression. The regression results are shown in Table 8. Columns (1), (2), and (3) represent the regression results of the East, Central, and West samples, respectively. The results show that the negative impact of AI input on the total demand for labor in the East and Central region samples is not significant. However, the AI input in the West region sample negatively affects the total demand for labor, and it is significant at the 5 % level. This indicates that the impact of AI inputs on the total demand for labor is directly related to the economic environment in which SMEs are located.

**Table 8:** Different regions and city types.

Variables	(1)	(2)	(3)	(4)	(5)	(6)
lnAI_inv	−0.008 (0.018)	−0.017 (0.024)	−0.019* (0.009)	0.014 (0.039)	−0.044** (0.017)	0.018 (0.028)
Controls	YES	YES	YES	YES	YES	YES
Enterprise fixed	YES	YES	YES	YES	YES	YES
Year fixed	YES	YES	YES	YES	YES	YES
Observations	525	60	50	185	335	115
F value	5.970***	61.540***	19.780***	4.430***	5.280***	4.700***
R^2^	0.265	0.475	0.805	0.333	0.467	0.356

According to existing research, the eastern region has the innate advantages of geographical location and the policy advantages of reform and opening up (Kong et al., 2020) [34], while the central region has a solid industrial foundation, many industrial categories, and regional computing power follows the eastern region. Therefore, it is located in the eastern and the development level and technical level of enterprises in the central region are far superior to those in the western region. However, on the one hand, the improvement in the technological development level in the eastern and central regions has gradually neutralized the substitution effect of AI by the creation effect. In the western region, the level of technological innovation is low, and the impact of small and micro enterprises on AI investment is still dominated by the substitution effect; On the other hand, the development trend in the eastern and central regions has attracted a large number of high-skilled talents, causing a part of the medium- and low-skilled labor force to move to the western region, and this part of the labor force faces a greater risk of being replaced.

#### 6.1.3 Different city types

Columns (4), (5) and (6) of Table 8 show the regression results of AI inputs on the total demand for labor in first-tier cities, new first-tier cities, and second-tier and lower cities, respectively. The results show that the positive impact of AI investment on total demand for labor in first-tier cities is not significant; AI investment in new first-tier cities has a significant negative impact on total demand for labor, with a significance level of 5 %; and AI investment in second-tier and lower cities has a non-significant positive impact on total demand for labor in enterprises. Among them, the reason why AI inputs in new first-tier cities have a substitution effect on the total demand for labor in enterprises is that most of the new first-tier cities are municipalities directly under the central government, regional center cities, provincial capitals in the eastern economically developed regions and coastal open cities, with rapid development of the private economy, and the application of new technologies such as AI has a significant inhibitory effect on the total demand for labor in the private sector, leading to the substitution effect of AI inputs on total demand for labor in the new first-tier cities.

#### 6.1.4 Different industries’ AI input

In order to examine the impact of different industries' AI inputs on enterprises’ total demand for labor, this paper selects the industries with the top five sample sizes (sample size ≥30) in the research sample for regression analysis. The results are shown in Table 9. Among them, column (1) is the regression result of the information transmission, software and information technology service industry, which shows that the negative impact of AI input on the total demand for labor is not significant; column (2) is the regression result of the manufacturing industry, which shows that the negative impact of AI input on the total demand for labor is not significant; and column (3) is the regression result of the wholesale and retail industry, which shows that AI input has a positive impact on the total demand for labor, and is significant at the 5 % level; column (4) is the regression results of the transportation, storage and postal industry, which shows that the negative impact of AI inputs on the total demand for labor is not significant; column (5) is the regression results of the accommodation and catering industry, which shows that the negative impact of AI inputs on the total demand for labor in enterprises is not significant; column (6) is the regression results of the service industry, which shows that the negative impact of AI inputs on the total demand for labor is insignificant. The regression results of the above industries show that AI input in the wholesale and retail industry has a significant promotion effect on the total demand for labor in enterprises, while the negative effect of AI input in other industries on the total demand for labor in enterprises is not significant.

**Table 9:** Different industries’ AI input.

Variables	(1)	(2)	(3)	(4)	(5)	(6)
lnAI_inv	−0.025 (0.027)	−0.022 (0.028)	0.032** (0.012)	−0.026 (0.019)	−0.018 (0.063)	−0.010 (0.016)
Controls	YES	YES	YES	YES	YES	YES
Enterprise fixed	YES	YES	YES	YES	YES	YES
Year fixed	YES	YES	YES	YES	YES	YES
Observations	140	140	100	60	30	455
F value	7.800***	24.010***	11.120**	22.870***	–	6.890***
R^2^	0.651	0.387	0.413	0.681	0.872	0.270

#### 6.1.5 The time length of AI application

For companies at different stages of artificial intelligence technology application, there are certain differences in the impact of artificial intelligence investment on the company's labor demand (Feng et al., 2024) [53]. In this paper, group regressions are conducted on the median duration of enterprises' application of AI technology (year - year of the start of application of AI technology), and the results are shown in Table 9. Columns (1) and (2) of Table 9 report the regression results of AI inputs on the total demand for labor for low (late) and high (early) enterprises' time of applying AI technology. The regression coefficient of AI inputs is significantly negative at the 1 % level in the regression of column (1) for the sample of the later application of AI technology, while the regression coefficient of column (2) is positive but not significant. The results indicate that the impact of AI inputs on the total demand for labor is mainly concentrated in enterprises that apply AI technology later.

#### 6.1.6 Labor intensity

We analyze the role of labor intensity in the impact of AI inputs on the total demand for labor by constructing a labor intensity indicator (Li and Zhao, 2020) [47], i.e., Labor Intensity = Employee Size/Main Business Revenue. Drawing on the grouping method of Cai et al. (2021) [46], the grouping regression is conducted with the median labor intensity, and the results are shown in Table 10. Columns (3) and (4) of Table 9 report the regression results of AI inputs on the aggregate demand for labor in low and high labor-intensive enterprises, respectively. Column (3) shows the regression results of the low labor-intensity sample, which shows that the regression coefficient of AI inputs is significantly negative at the 10 % level; and the regression coefficient of AI inputs in the high-labor-intensity sample in column (4) is not significant. The results show that the impact of AI inputs on the total demand for enterprise labor is mainly concentrated in low labor-intensive enterprises, i.e., the impact of AI inputs on the total demand for enterprise labor is directly related to the changes in labor factors.

**Table 10:** The time length of AI application and labor intensity.

Variables	(1)	(2)	(3)	(4)
lnAI_inv	−0.040*** (0.015)	0.088 (0.100)	−0.031* (0.017)	0.017 (0.029)
Controls	YES	YES	YES	YES
enterprise fixed	YES	YES	YES	YES
Year fixed	YES	YES	YES	YES
Observations	376	259	327	308
F value	7.150***	12.270***	6.320**	3.720***
R^2^	0.431	0.408	0.442	0.295

### 6.2 Mechanism analysis

The analysis reveals that the impact of AI inputs on the total demand for labor is not significant, which is consistent with the conclusion reached by Südekum et al. (2017) [8]. However, its finding that AI reduces the demand for labor in the manufacturing industry and increases the demand for labor in the service industry is inconsistent with the results reached in this paper, i.e., the inputs of AI do not significantly reduce the demand for labor in the manufacturing industry as well as in the service industry but rather increased the labor demand in the wholesale and retail industry. In order to further analyze the mechanism of the role of AI inputs on the total demand for labor in enterprises, this paper carries out a mechanism analysis of the impact of AI inputs on the total demand for labor in enterprises in terms of the substitution effect of AI on employment, the creation effect (Acemoglu and Restrepo, 2017; Cao and Zhou, 2018) [48,49], and the complementary effect (Yu et al., 2019)[50].

#### 6.2.1 Low-skilled labor demand

This paper takes the number of employees with less than a bachelor's degree as an explanatory variable and conducts regression analysis of AI inputs to examine the impact of AI inputs on the demand for low-skilled labor in enterprises. The results are shown in Table 11. Among them, column (1) shows the regression results for the whole sample, indicating that AI input has a significant substitution effect on low-skilled labor. Columns (2) and (3) show the regression results for the samples of non-state-owned enterprises and state-owned enterprises, respectively. The results indicate that the effect of AI input on the low-skilled labor force of the enterprises is significantly negative in non-state-owned enterprises and insignificant in state-owned enterprises. Columns (4) and (5) show the regression results for the samples of non-private enterprises and private enterprises, respectively. The results indicate that AI inputs have a significant substitution effect on low-skilled labor in private enterprises, and the effect is not significant in non-private enterprises. Columns (6) and (7) are regression results for the samples of non-high-tech enterprises and high-tech enterprises, respectively. The results indicate that the substitution effect of AI inputs on low-skilled labor is significantly in high-tech enterprises, and in non-high-tech enterprises is not significant.

**Table 11:** Low-skilled labor demand.

Variables	(1)	(2)	(3)	(4)	(5)	(6)	(7)
lnAI_inv	−0.036* (0.021)	−0.052** (0.022)	−0.007 (0.057)	0.028 (0.060)	−0.058*** (0.021)	−0.015 (0.022)	−0.095* (0.049)
Controls	YES	YES	YES	YES	YES	YES	YES
Enterprise fixed	YES	YES	YES	YES	YES	YES	YES
Year fixed	YES	YES	YES	YES	YES	YES	YES
Observations	635	550	85	125	510	450	185
F value	2.980***	3.130***	1.540	1.120	3.090***	4.520***	2.400**
R^2^	0.092	0.119	0.183	0.171	0.116	0.089	0.243

#### 6.2.2 Impact of AI inputs on the demand for high-skilled labor

The number of employees with a bachelor's degree or higher is used as the explanatory variable, and regression analysis of AI input is conducted to examine the effect of AI input on the demand for high-skilled labor. The results are shown in Table 12. Column (1) shows the impact of the whole sample, and the results indicate that the impact of AI inputs on the high-skilled labor of enterprises is not significant. Columns (2) and (3) show the regression results of the sample of non-state-owned enterprises and state-owned enterprises, respectively. The results indicate that the effect of AI inputs on the demand for high-skilled labor of enterprises is not significant in both non-state-owned enterprises and state-owned enterprises. Columns (4) and (5) are the regression results of the samples of non-private enterprises and private enterprises, respectively. The results show that the impact of AI inputs on the demand for high-skilled labor is insignificant in both non-private enterprises and private enterprises. Columns (6) and (7) are the regression results of the samples of non-high-tech enterprises and high-tech enterprises, respectively. The results show that the effect of AI inputs on the demand for highly skilled labor is significantly positive in the non-high-tech enterprises and insignificant in the high-tech enterprises.

**Table 12:** High-skilled labor.

Variables	(1)	(2)	(3)	(4)	(5)	(6)	(7)
lnAI_inv	0.015 (0.014)	0.012 (0.016)	−0.003 (0.029)	0.001 (0.024)	0.013 (0.016)	0.041** (0.016)	−0.024 (0.022)
Controls	YES	YES	YES	YES	YES	YES	YES
Enterprise fixed	YES	YES	YES	YES	YES	YES	YES
Year fixed	YES	YES	YES	YES	YES	YES	YES
Observations	635	550	85	125	510	450	185
F value	18.960***	17.890***	4.060***	4.650***	17.270***	13.500***	10.610***
R^2^	0.478	0.510	0.463	0.398	0.511	0.442	0.687

#### 6.2.3 The creation effect of AI input on labor demand

The number of AI-related employees is used as the explanatory variable, and regression analysis of AI inputs is conducted to examine the effect of AI inputs on enterprise AI labor demand. The results are shown in Table 13. Column (1) shows the regression results of the whole sample, and the results indicate that the creation effect of AI input on enterprise labor demand is significantly positive. Columns (2) and (3) show the regression results of the samples of non-state-owned enterprises and state-owned enterprises, respectively. The results indicate that AI inputs have a significant role in promoting the demand for AI labor in both non-state-owned enterprises and state-owned enterprises. Columns (4) and (5) show the regression results of the samples of non-private enterprises and private enterprises, respectively. The results indicate that AI inputs have a significant role in promoting the demand for AI labor in both non-private enterprises and private enterprises. Columns (6) and (7) show the regression results of the samples of non-high-tech enterprises and high-tech enterprises, respectively. The results indicate that AI inputs have a significant role in promoting the demand for AI labor in both non-high-tech enterprises and high-tech enterprises.

**Table 13:** The creation effect of AI input on the demand for AI labor in enterprises.

Variables	(1)	(2)	(3)	(4)	(5)	(6)	(7)
lnAI_inv	0.351*** (0.059)	0.363*** (0.064)	0.225* (0.126)	0.268** (0.121)	0.360*** (0.064)	0.291*** (0.068)	0.570*** (0.098)
Controls	YES	YES	YES	YES	YES	YES	YES
Enterprise fixed	YES	YES	YES	YES	YES	YES	YES
Year fixed	YES	YES	YES	YES	YES	YES	YES
Observations	635	550	85	125	510	450	185
F value	26.320***	25.050***	13.130***	3.550***	25.290***	23.590***	9.440***
R^2^	0.633	0.638	0.661	0.551	0.649	0.634	0.713

## 7 Implications

### 7.1 Theoretical implications

Through applying task model and two-way fixed effects model, the current research offers several crucial contributions to the comprehension of AI investment in the context of SMEs in China. To the best of our knowledge, this is the first study that uses the SMEs to examine the effect of AI investment on labor demand. Consequently, this constitutes an essential addition to the emerging knowledge on AI investment in the existing literature [4,6,12].

Unlike the existing literature, this paper is an important addition to the existing literature, which mostly samples data from listed companies, by using data from micro and small firms to study the impact of AI on labor demand. In addition, the basic results of this paper also differ from the existing literature in that the results of this paper are non-significant, whereas the existing literature has either a negative or a positive impact.

The research in this paper shows that the impact of AI inputs on the total labor demand of Chinese SMEs is not too obvious, but it shows a significant negative impact in non-state-owned enterprises, private enterprises, and high-tech enterprises. The substitution effect on low-skilled labor has already appeared, especially in private enterprises and high-tech enterprises. For China, the application of AI has entered a stage of rapid development. With the deepening of the degree of intelligence of enterprises, the substitution effect of AI technology on low-skilled labor will continue to increase in the total demand. In the long run, this seems to lead to a decline in the total demand for labor in enterprises, resulting in the consequences of technological unemployment (Han and Han, 2020; Wang et al., 2017) [30,51]. Therefore, it is necessary to plan well in advance to avoid the impact of AI on employment. In this regard, in addition to drawing on the policy experience of Nordic countries in providing short-term unemployment insurance and retraining opportunities for the unemployed (Wang and Dong, 2020) [52], the government should encourage enterprises to provide low-skilled laborers with “learning-by-doing” training. This would upgrade the new technology level of low-skilled laborers and increase government subsidies for enterprise training, so that the skill transformation of replaced employees can be completed smoothly.

Research results show that a certain proportion of AI-related employees are low-skilled laborers, which indicates that there is a skill conversion in the demand for low-skilled laborers. “Learning by doing” training allows enterprises to save the increase in human capital brought about by hiring employees with AI-related experience. At the same time, it avoids the labor productivity uncertainty brought about by new employees adapting to the enterprise environment. The government gains the return of stable employment. The research in this paper shows that the application of AI technology in non-state-owned enterprises shows more significant labor substitution effects. With the development of AI technology, migrant workers and other high-unemployment-risk groups will inevitably face a greater impact. Therefore, drawing on international beneficial experience, it is necessary to improve the design of the unemployment insurance system, increase the coverage and effectiveness of unemployment insurance, and strengthen the social security of informal work. This can play a positive role in realizing higher quality and fuller employment in the era of AI.

The application of AI technology, while substituting for low-skilled labor, also creates new job opportunities and increases the demand of enterprises for AI-related personnel. This demand does not differ by region, city, and type of enterprise, but there are obvious industry differences. Therefore, the relevant employment training system and re-employment policies should be further improved to enhance the adaptability of workers with different skills to the new economy. The talent training system should be further optimized to strengthen the cultivation of professionals and “complementary” talents in AI and other related fields, so as to seize the development opportunities brought about by the new round of technological revolution. Questionnaire research results show that after leaving the original position, employees are mainly engaged in the occupation of self-employment, professional and technical personnel, and clerical staff (the top three). Therefore, the local government, in promoting the application of AI, should at the same time provide entrepreneurial counseling and policy support for employees leaving the job, skills training, and other social security measures. This can cushion the impact of AI on employment and improve the competitiveness of the secondary employment of the job.

The substitution effect of AI inputs on labor force is more significant in enterprises with low labor intensity and short application time of AI technology. Therefore, promoting the upgrading of the industrial structure of enterprises and accelerating the digital transformation of enterprises can help create more employment opportunities and jobs and realize the high-quality development of China's economy.

## 8 Conclusions and recommendations

This paper empirically examines the impact of AI inputs on enterprise labor demand in Chinese MSMEs, using a two-way fixed-effects model. The empirical results show that the negative effect of AI inputs on the total demand for labor in enterprises is not significant, but this negative effect is significant in non-state-owned enterprises, private enterprises, and high-tech enterprises, in the western region, in the new first-tier cities, as well as in enterprises with a shorter period of AI use and low labor intensity, and it shows a significant difference at different levels. In addition, the effect of AI input on the total demand for labor in enterprises is significantly positive in the wholesale and retail industry. The mechanism analysis shows that the substitution effect of AI inputs on low-skilled labor demand is significant, and this effect exists in non-state-owned enterprises, private enterprises, and high-tech enterprises at the same time, but there is no regional, city type, or industry heterogeneity. The complementary effect of AI inputs on high-skilled labor demand exists only in non-high-tech enterprises, and in enterprises of the wholesale and retail industry, and has a significant impact on the total demand for labor in the service industry other than the wholesale and retail industry. In short, the impact of AI inputs on the total demand for enterprise labor is insignificant due to the offsetting of the substitution effect and the complementary effect as well as the creation effect. With the enhancement of AI inputs, the expansion of the production scale of enterprises as well as the improvement of labor productivity ultimately lead to the continuous optimization of the labor structure of enterprises, and thus there is a potential risk of a decline in the demand for labor in general. With the enhancement of AI inputs, the expansion of the production scale of enterprises as well as the improvement of labor productivity ultimately lead to the continuous optimization of the labor structure of enterprises, and thus there is a potential risk of a decline in the demand for labor in general. The conclusions of this paper provide empirical evidence for a comprehensive understanding of the impact of the application of AI technology on labor demand and the formulation of corresponding policies, with important policy implications:

Firstly, the research in this paper shows that the impact of AI inputs on the total labor demand of Chinese micro and small enterprises is not too obvious, but it shows a significant negative impact in non-state-owned enterprises, private enterprises, and high-tech enterprises. The substitution effect on low-skilled labor has already appeared, especially in private enterprises and high-tech enterprises. For China, the application of AI has entered a stage of rapid development. With the deepening of the degree of intelligence of enterprises, the substitution effect of AI technology on low-skilled labor will continue to increase in the total demand. In the long run, this seems to lead to a decline in the total demand for labor in enterprises, resulting in the consequences of technological unemployment (Han and Han, 2020; Wang et al., 2017) [30,51]. Therefore, it is necessary to plan well in advance to avoid the impact of artificial intelligence on employment. In this regard, in addition to drawing on the policy experience of Nordic countries in providing short-term unemployment insurance and retraining opportunities for the unemployed (Wang and Dong, 2020) [52], the government should encourage enterprises to provide low-skilled laborers with “learning-by-doing” training. This would upgrade the new technology level of low-skilled laborers and increase government subsidies for enterprise training, so that the skill transformation of replaced employees can be completed smoothly. Research results show that a certain proportion of AI-related employees are low-skilled laborers, which indicates that there is a skill conversion in the demand for low-skilled laborers. “Learning by doing” training allows enterprises to save the increase in human capital brought about by hiring employees with AI-related experience. At the same time, it avoids the labor productivity uncertainty brought about by new employees adapting to the enterprise environment. The government gains the return of stable employment. The research in this paper shows that the application of AI technology in non-state-owned enterprises shows more significant labor substitution effects. With the development of AI technology, migrant workers and other high-unemployment-risk groups will inevitably face a greater impact. Therefore, drawing on international beneficial experience, it is necessary to improve the design of the unemployment insurance system, increase the coverage and effectiveness of unemployment insurance, and strengthen the social security of informal work. This can play a positive role in realizing higher quality and fuller employment in the era of artificial intelligence.

Secondly, the research in this paper finds that the application of AI technology, while substituting for low-skilled labor, also creates new job opportunities and increases the demand of enterprises for AI-related personnel. This demand does not differ by region, city, and type of enterprise, but there are obvious industry differences. Therefore, the relevant employment training system and re-employment policies should be further improved to enhance the adaptability of workers with different skills to the new economy. The talent training system should be further optimized to strengthen the cultivation of professionals and “complementary” talents in AI and other related fields, so as to seize the development opportunities brought about by the new round of technological revolution. Questionnaire research results show that after leaving the original position, employees are mainly engaged in the occupation of self-employment, professional and technical personnel, and clerical staff (the top three). Therefore, the local government, in promoting the application of artificial intelligence, should at the same time provide entrepreneurial counseling and policy support for employees leaving the job, skills training, and other social security measures. This can cushion the impact of artificial intelligence on employment and improve the competitiveness of the secondary employment of the job.

Thirdly, the research in this paper shows that the substitution effect of AI inputs on labor force is more significant in enterprises with low labor intensity and short application time of AI technology. Therefore, promoting the upgrading of the industrial structure of enterprises and accelerating the digital transformation of enterprises can help create more employment opportunities and jobs and realize the high-quality development of China's economy.

## Funding statement

The research received no specific grant from funding agencies in the public, commercial, or not-for-profit sectors.

## Ethics declarations

This study was reviewed and approved by the ethical committee at Capital University of Economics and Business.

## Informed consent statement

Informed consent was obtained from all participants before the data was collected. Participants were informed about their rights, the purpose of the study and to safeguard their personal information.

## Data availability statement

Data will be made available upon the request and could be accessed by contacting the corresponding author.

## CRediT authorship contribution statement

**Gan Xu:** Writing – original draft, Investigation, Formal analysis. **Yue Qiu:** Methodology, Investigation, Data curation. **Jingyu Qi:** Writing – review & editing, Project administration, Conceptualization.

## Declaration of competing interest

The authors declare that they have no known competing financial interests or personal relationships that could have appeared to influence the work reported in this paper.

## Footnotes

1 O*NET has detailed descriptions of the world of work for use by job seekers, workforce development and HR professionals, students, developers, researchers, and more! It is sponsored by the U.S. Department of Labor, Employment & Training Administration, and developed by the National Center for O*NET Development.

2 The concept of SMEs comes from the Ministry of Industry and Information Technology's “Provisions on the Standard Regulations for the Delineation of Small and Medium-sized Enterprises”, and the specific standards are formulated based on the indicators of the enterprise's employees, business revenue, total assets and other indicators in combination with the industry characteristics.
